# Pin1 Promotes NLRP3 Inflammasome Activation by Phosphorylation of p38 MAPK Pathway in Septic Shock

**DOI:** 10.3389/fimmu.2021.620238

**Published:** 2021-02-25

**Authors:** Ruijie Dong, Zhenyi Xue, Guangyue Fan, Na Zhang, Chengzhi Wang, Guangliang Li, Yurong Da

**Affiliations:** ^1^ Department of Immunology, Key Laboratory of Immune Microenvironment and Diseases of Educational Ministry of China, School of Basic Sciences, Tianjin Medical University, Tianjin, China; ^2^ Department of Cardiology, Tianjin Medical University General Hospital, Tianjin, China

**Keywords:** Pin1, NLRP3 inflammasome, p38 MAPK, macrophages, septic shock

## Abstract

Pin1 is the only known peptidyl-prolyl cis-trans isomerase (PPIase) that can specifically recognize and isomerize the phosphorylated Serine/Threonine-Proline (pSer/Thr-Pro) motif, change the conformation of proteins through protein phosphorylation, thus regulate various cellular processes in the body. Pin1 plays an important role in cancer, Alzheimer’s disease, and autoimmune diseases. However, the specific mechanism of Pin1 regulation in LPS-induced septic shock is unclear. Here, we found that lack of Pin1 reduced shock mortality and organ damage in mice, and NLRP3 inflammasome activation also was reduced in this process. We further confirmed that Pin1 can affect the expression of NLRP3, ASC, Caspase1, and this process can be regulated through the p38 MAPK pathway. We analyzed that p38 MAPK signaling pathway was highly expressed in septic shock and showed a positive correlation with Pin1 in the Gene Expression Omnibus database. We found that Pin1 could affect the phosphorylation of p38 MAPK, have no obvious difference in extracellular signal-regulated kinases (ERK) and Jun-amino-terminal kinase (JNK) signaling. We further found that Pin1 and p-p38 MAPK interacted, but not directly. In addition, Pin1 deficiency inhibited the cleavage of gasdermin D (GSDMD) and promoted the death of macrophages with LPS treatment, and reduced secretion of inflammatory cytokines including IL-1β and IL-18. In general, our results suggest that Pin1 regulates the NLRP3 inflammasome activation by p38 MAPK signaling pathway in macrophages. Thus, Pin1 may be a potential target for the treatment of inflammatory diseases such as septic shock.

## Introduction

Sepsis is the leading cause of death in clinically infected patients, especially in medical intensive care units (1). Generally speaking, inflammation is the body’s normal defense against the invasion of microorganisms. However, excessive inflammation, such as sepsis, may cause tissue and organ damage and even death (2–6). The pro-inflammatory cytokines IL-1β and IL-18, mainly from macrophages, play important role in inflammatory diseases. The biological activation of IL-1β and IL-18 are usually result of the inactive precursors (pro-IL-1β and pro-IL-18) cleaved by the intracellular cysteine-aspartic protease caspase-1. This process can be regulated by the inflammasome (1, 7, 8).

Inflammasome plays important role in host defense (9, 10). Inflammasome may be involved in the pathogenesis of many human inflammatory diseases such as gout, diabetes, atherosclerosis and coloretal cancer (11). Nucleotide binding domain, leucine-rich repeat-containing receptor (NLR) family protein 3 (NLRP3) is essential for the identification of pathogen-associated molecular patterns (PAMPs) or damage-associated molecular patterns (DAMPs) (12). NLRP3 serves as an inflammasome sensor, which has been widely studied (11). A key component of NLRP3 interacts closely with apoptosis-associated speck-like protein (ASC) to recruit the precursor form of Caspase-1, then forming the multi-component protein complex–NLRP3 inflammasome (1, 13, 14). NLRP3 inflammasome is an important component of innate immunity and plays an important role in inflammatory responses (12). Regarding the activation of NLRP3 inflammasome, two signal models have been proposed. The initiating signal coming from stimulating pathogen recognition receptors (PRRs) such as NLRs and Toll-like receptors (TLRs), which can activate nuclear factor κB (NF-κB) and mitogen-activated proliferation protein kinase (MAPK) pathways that up -regulate transcription of the NLRP3 inflammasome component. The second signal is caused by various factors, such as ATP, pore formation, potassium (K^+^) outflow, lysosomal instability/rupture, and mitochondrial reactive oxygen species (mtROS) (7, 15).

After activation, caspase-1 cleaves pro-IL-1β and pro-IL-18 to produce biologically active IL-1β and IL-18 promotes inflammation or induces the death of inflammatory cells that is called “pyroptosis” (7, 8). Caspase-1 can specifically recognize and cleave gasdermin D (GSDMD), which is the key event of pyroptosis (8, 16). The divided GSDMD can form small pore in the cell membrane to release inflammatory factors to the outside of the cell that causing inflammation (17, 18).

Pin1, a member of the peptide-proline isomerase family, is the only known enzyme that can specifically bind the phosphorylated Serine/Threonine-Proline (pSer/Thr-Pro) to efficiently catalyze the peptide bond cis - trans isomerism (19–21). Pin1 significantly accelerates cis-trans isomerism, depending on specific targets and local structural constraints, thereby regulating the phosphorylated conformation of proteins between two different structures and resulting in changes of protein function (22). Pin1 can regulate various cell activities such as cell proliferation, apoptosis, embryonic development, and neurons differentiation, stress response and immune regulation, and are involved in the occurrence and development of various diseases such as cancer, autoimmune diseases, Alzheimer’s disease, brain injury, and aging (23–26). However, whether Pin1 can control the transcription and activation of NLRP3 inflammasome components is still unclear.

Here, we show that Pin1^-/-^ mice can reduce the mortality of LPS-induced mice. In this process, Pin1 regulate the transcription and activation of NLRP3 inflammasome by regulating the phosphorylation of p38 MAPK signaling pathway, thereby affecting the inflammatory response. These findings indicate that Pin1 plays an important role in the inflammatory response induced by LPS.

## Material and Methods

### Animals

C57BL/6 mice (6–8 weeks old) were ordered from the Academy of Military Medical Science (Beijing, China). Our Pin1 knockout mouse was authorized by Professor Lu Kunping of Harvard Medical School and provided by Institution of translational medicine of Fujian Medical University. All animals were raised in a specific pathogen-free animal environment at the Experimental Animal Center of Tianjin Medical University (Tianjin, China). The experiments were approved by the Animal Ethics Committee of Tianjin Medical University (Tianjin, China) and were conducted in accordance with the guidelines for animal care. The standard PCR method was used to identify the gene of mice.

### Reagents

Lipopolysaccharide (LPS; E. coli O111:B4, L3024), ATP and nigericin were from Sigma-Aldrich (St. Louis, USA), Enzyme-free cell digestion fluid (Applygen, Beijing), siRNA Transfection Reagent (Polyplus Transfection, France), Murine M-CSF (315-02, Peprotech), protein G (invitrogen by Thermo Fisher Scientific), Glutathione Sepharose ™ 4B (GE Healthcare, USA).

### Cytokine Analysis

Cell culture supernatants were measured for murine IL-1β and IL-18, IL-6, and TNF-α or human IL-1β and IL-18 with ELISA kits (Shanghai Meilian, Shanghai, China), Cell viability and cell lysis were measured with an LDH assay performed using CytoTox 96 Non-Radioactive Cytotoxicity Assay kit (Promega, USA).

### Cell Culture

Preparation of bone-marrow derived macrophages: Bone marrow cells were first extracted from femur and tibia of wild-type and Pin1^-/-^ mice, and then cultured in a 10cm culture dish at 2◊10^6 cells/ml, followed by 10 ng/ml M-CSF supplement in DMEM (10% serum) medium containing bone marrow cells. The cells were cultured in a carbon dioxide incubator for 6 days and supplemented with a fresh medium on the third day. On the sixth day, cells were collected for experiments. Macrophages were stimulated with 500 ng/ml LPS for 4 h, followed by stimulation with 5mM ATP and 20 uM nigericin for 30 min. RAW 264.7 cell and THP-1 cells (human mononuclear macrophage line) were purchased from the Cell Resource Center of Peking Union Medical College, which were cultured in DMEM containing 10% inactivated fetal bovine and antimicrobial agents (100 IU/ml penicillin,100 µg/ml streptomycin), and RPMI 1640 medium containing 10% fetal bovine serum and antimicrobial agents (100 IU/ml penicillin, 100 µg/ml streptomycin), respectively.

### Small Interfering RNAs and Transfection

Control siRNA and siPin1 were synthesized by RiboBio (Guangzhou, China). The mouse siRNA and human siRNA for Pin1 knockdown had the following sequence: 5′- GCUCAGGCCGGGUGUACUA -3′ (sense sequence) and 5′- UCAGGCCGAGUGUACUACUdTdT -3′ (sense sequence), respectively. According to the manufacturer’s instructions, cells were treated with siRNAs (final concentration of 25 nM) and harvested 48 h after siRNA treatment using Lipofectamine RNAiMAX (Invitrogen, Carlsbad, CA, USA).

### Quantitative Real-Time PCR

RNA was extracted using Trizol reagent (Invitrogen, Carlsbad, USA) according to manufacturer’s instructions. After RNA purification, DNA enzymes were used to remove contaminated genomic DNA from the samples. Random hexamers and M-MLV reverse transcriptase (Promega, Madison, USA) were used for RNA reverse transcription. All other reverse transcription reagents were purchased from Takara (Takara, Japan). The gene-specific primers were synthesized from Genewiz (Suzhou, China). SYBR Green mix (Takara, Japan) was used for relative quantitative real-time PCR under the guidance of the manufacturer’s instructions. The reaction was run on an ABI PRISM 7500 Fast Real-Time PCR System (Applied Biosystems Inc., Foster City, California, USA), and repeated in triplicate. The results were analyzed using ABI 7500 software (Version 2.0.5). The primer were used as following: mouse *Nlrp3* forward, 5′-ATTACCCGCCCGAGAAAGG-3′ and reverse, 5′-TCGCAGCAAAGATCCACACAG-3′; mouse *Asc* forward, 5′-CTTGTCAGGGGATGAACTCAAAA-3′ and reverse, 5′-GCCATACGACTCCAGATAGTAGC-3′; mouse *Casp1* forward, 5′-ACAAGGCACGGGACCTATG-3′ and reverse, 5′-TCCCAGTCAGTCCTGGAAATG-3′; mouse *IL-1β* forward, 5′-GCAACTGTTCCTGAACTCAACT-3′ and reverse, 5′-ATCTTTTGGGGTCCGTCAACT-3′; mouse *Gapdh* forward, 5’-GCACCGTCAAGGCTGAGAAC-3′ and reverse 5′-TGGTGAAGACGCCAGTGGA -3′.

### Western Blot Analysis

Cells were lysed using Lysis buffer containing 10 mM Tris-buffer (pH 7.6), 1%Triton X-100, 1% phosphatase inhibitor cocktail and 1 mM PMSF. Then, cell lysates were boiled in SDS sample buffer and resolved on a 10% SDS-PAGE gel. Proteins were transferred onto PVDF membranes and incubated overnight with primary antibodies against using rabbit anti-p-p38MAPK (Cat# 4511, RRID : AB_2139682), p38MAPK(Cat# 8690, RRID : AB_10999090), p-ERK1/2(Cat# 4370, RRID : AB_2315112), ERK1/2(Cat# 4695, RRID : AB_390779), p-JNK(Cat# 4668, RRID : AB_823588) and JNK(Cat# 9252, RRID : AB_2250373) antibodies are from Cell Signaling Technology, anti-Pin1 (Cat# 10495-1-AP, RRID : AB_2163943, Proteintech), NLRP3 (Cat# 19771-1-AP, RRID : AB_10646484, Proteintech), ASC (Cat# AG-25B-0006, RRID : AB_2490440, Adipogen), GSDMD (Cat# ab209845, RRID : AB_2783550, Abcam), GSDMDC1(Cat# sc-81868, RRID : AB_2263768, Santa Cruz Biotechnology), caspase-1(p20)(mouse) (Cat# AG-20B-0042-C100, RRID : AB_2755041, Adipogen), caspase-1(p20)(human) (Cat# AG-20B-0048-C100, RRID : AB_2490257, Adipogen), and GAPDH (Cat# 10494-1-AP, RRID : AB_2263076, Proteintech), Anti-rabbit IgG HRP-linked Antibody(Cat# 7074, RRID : AB_2099233, Cell Signaling Technology) and Anti-mouse IgG HRP-linked Antibody(Cat# 7076, RRID : AB_330924, Cell Signaling Technology), IgG (Cat# sc-2027, RRID : AB_737197, Santa Cruz Biotechnology). Immunoblots were examined by ECL detection reagent (Millipore Corporation, Billerica, MA, USA).

### Co-Immunoprecipitation Assay

Extract mouse bone marrow and add cytokines to induce bone marrow macrophages, the cells were harvested and lysed with lysis buffer (150 mM Tris -Hcl,50 mM Nacl,0.3%NP-40, 2 mM EDTA) for 30 min, and the supernatant was collected after centrifugation. 1–2 μg of indicated primary antibody was added into supernatant to incubate overnight at 4 °C. The next day, protein G beads were added. And 3 h later, buffer was used for washing the beads three times, 5 min each time, then the beads were collected by centrifugation. Finally, samples were obtained by boiling in 2◊SDS loading buffer at 95°C for 10 min.

### Lentiviral Constructs and Infection

The Pin1 control and overexpressed plasmid was synthesized by the company (Huashengyuan, Tianjin, China). Pin1 plasmid was transfected into HEK293T cells with the pack-aging vectors psPAX2 (Addgene plasmid 12260) and pMD2.G (Addgene plasmid 12259) using PEI (Polyscience). After 48 and 72 h, the culture medium was collected and centrifuged at 2000g for 5 min to remove the cell debris *via* sedimentation, and the supernatant was filtered through a membrane. The samples were subsequently placed in 50-ml ultracentrifugation tubes, 5 x PEG 8000 was added as 1/4 volume of the supernatant, and the mixture was incubated overnight at 4 °C. The next day, the mixture was centrifuged at 4,000g for 30 min at 4 °C and resuspended for virus precipitation with ice-cold sterile PBS to collect LV-Pin1-GFP-Puro virus. The negative control viruses LV-control-GFP-Puro viruses were obtained similarly.

### GST Pulldown Assay and Dot Blot

The GST-vector and GST-Pin1plasmid (Huashengyuan, Tianjin, China) are transformed in BL21 bacteria, amplify the bacterial solution in a 37°C shaker to an OD600 of 0.6-0.8, add IPTG (0.1 mMol/ml), and cultivate overnight in a 16°C shaker. The next day, the bacterial solution was centrifuged at 500g for 10 min at 4 °C and resuspended for bacterial precipitation with ice-cold lysis buffer (50 mM Tris-Hcl,150mM Nacl,0.05%NP-40, PH=7.5). Next, sonicate the bacteria liquid on ice until the bacteria become clear. The supernatant was collected by 4000g for 15 min at 4 °C. Add an appropriate amount of GST-4B beads pre-washed with cold PBS to the supernatant, and incubate at room temperature for 1 h. The beads were collected by 500g for 5 min at 4 °C. Wash the beads three times with cold PBS. Then add p38 phosphopeptide (TDDEMT(p)GYVATRW, Chutai, Shanghai), which was phosphorylated at threonine 180, T180(p), to the beads and incubate overnight at 4°C. The next day, wash the beads with PBS three times, and then wash the beads with the reduced glutathione eluent (30.73 mg of glutathione is dissolved in 10 mL of 50 mM Tris, PH=8.0). At this time, the eluent is collected. Spot the sample on the PVDF or NC membrane with the eluent. After it is air-dried, the 5% skimmed milk powder is blocked for 1 h, and the primary antibody is incubated overnight. Immunoblots were examined by ECL detection reagent.

### Flow Cytometry

After 12 h of intraperitoneal injection of LPS in mice, the peritoneal lavage fluid was extracted, and cells were washed with PBS containing 1%FBS. Anti-CD11b (Cat# 101205, RRID : AB_312788), anti-MHC II (Cat# 107607, RRID : AB_313322) and anti-CD206 (Cat# 141707, RRID : AB_10896057) antibodies with fluorochromes were stained according to the instructions. Isotype antibody controls were used to monitored nonspecific staining. All antibodies were obtained from Biolegend. The data were obtained on a FACSCanto II flow cytometer (BD Biosciences, USA) and analyzed by FlowJo software (Tree star, Ashland, OR).

### Microscopy Imaging of Cell Death

To observe the death morphology of cells, the cells were spread in a 12-well plate and treated according to the instructions of living cell static imaging. Static bright-field images of pyroptotic cells were captured on an Olympus CKX53 microscope. All displayed image data represents at least three randomly selected regions.

### Histopathology

Liver, spleen, and kidney tissues from LPS-treated (12 h) WT mice and Pin1^-/-^ mice through cardiac perfusion with 4% paraformaldehyde, then dissected and post-fixed overnight. These tissues were embedded in paraffin and sectioned 6 µm for hematoxylin and eosin staining(H&E). The degree of infiltration of inflammatory cells was analyzed and quantified by routine histology and ImageJ software, respectively.

### Septic Shock Model

Mice at 8 weeks old were intraperitoneally injected with LPS to induce the secretion of inflammatory cytokines. After 12 h of injection, 4ml of PBS containing 1%FBS was injected into the peritoneal cavity of mice and the fluid was collected. The expressions of IL-18 and IL-1β in mice peritoneal lavage fluid were detected by ELISA. The healthy mice were observed at specific intervals after LPS injection into the peritoneal cavity of mice for inducing septic shock.

### Statistics

In a representative experiment, the data were expressed as mean ± SD of the three measurements. All statistical tests were analyzed by 2-tailed Student’s t test for comparison of 2 groups and ANOVA using GraphPad Prism 8.0 software (GraphPad Software, San Diego, USA) for comparison of multiple groups. P<0.05 was considered statistically significant. Survival was analyzed with the log-rank test. P<0.05 was considered statistically significant.

## Results

### Pin1 Deficiency Relieves LPS-Induced Septic Shock and Organ Damage

**Figure 1:**
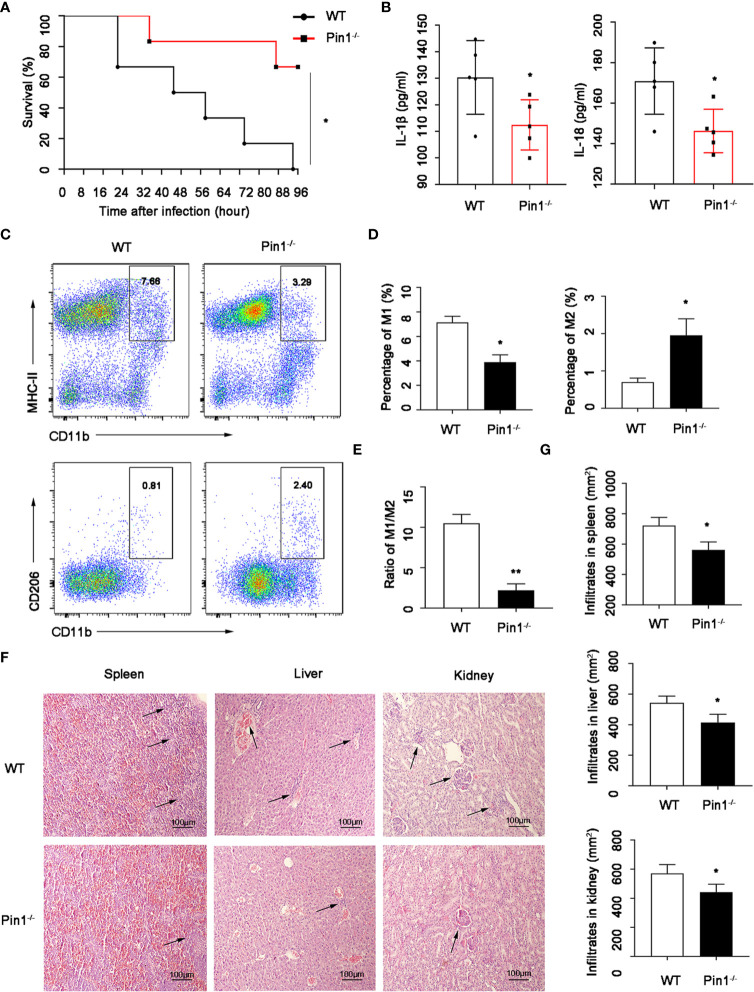
Pin1 deficiency relieves lipopolysaccharide (LPS)-induced septic shock and organ damage. **(A)** Survival of WT mice (n=12) and Pin1^-/-^ mice (n=12) infected intraperitoneally with LPS (30 mg/kg of body weight). **(B)** The secretion of IL-18 and IL-1β in the peritoneal lavage fluid of WT mice (n=5) and Pin1^-/-^ mice (n =5) after intraperitoneal injection of LPS (30 mg/kg of body weight). **(C)** After intraperitoneal injection of LPS in WT mice (n=5) and Pin1^-/-^ mice (n =5), the proportions of M1 and M2 macrophages in the abdominal fluid were analyzed by flow cytometry. **(D)** Quantitative analysis of the percentages of M1 and M2 macrophages in the abdominal fluid of WT mice (n=5) and Pin1^-/-^ mice (n=5) treated with LPS. **(E)** The ratio of M1 and M2 macrophages in WT mice (n =5) and Pin1^-/-^ mice (n=5) treated intraperitoneally with LPS. **(F)** Histological analyses of spleens, livers, and kidneys from WT mice and Pin1^-/-^ mice. **(G)** Quantitative analysis of spleens, livers, and kidneys from WT mice and Pin1^-/-^ mice. The data represent the mean ± SD of one among three biological replicates, with three technical replicates each. *P < 0.05; **P < 0.01, log-rank test **(A)**, Student’s t test **(B, D, E, G)**.

To determine the potential role of Pin1 in LPS-induced septic shock pathogenesis, we treated mice with LPS in wild type (WT) and Pin1 knockout (Pin1^-/-^) groups to get septic shock mouse model, respectively. We observed a lower mortality rate in mice with septic shock induced by intraperitoneal injection in the Pin1^-/-^group than in the wild-type group (**Figure 1A**).

In addition, we detected macrophages in the peritoneal fluid of mice, and found that the expression of inflammatory factors IL-18 and IL-1β detected by peritoneal fluid was also obviously decreased (**Figure 1B**). Moreover, the M1-type macrophages (M1) in Pin1^-/-^ group were lower than those in the WT group, while M2-type macrophages (M2) were higher than those in the WT group (**Figures 1C, D** **)**, and the proportion of M1/M2 macrophages was significantly decreased (**Figure 1E**). We also detected the damage to the internal organs of mice by HE staining, and found that the spleen, liver, and kidney were less damaged and inflammatory cells were less infiltrated in Pin1^-/-^mice (**Figures 1F, G** **)**. These results suggest that Pin1 plays an important role in LPS-induced inflammatory response.

### Caspase-1 Activation and IL-1β and IL-18 Secretion in Macrophages Were Inhibited in Pin1^-/-^ Mice

**Figure 2:**
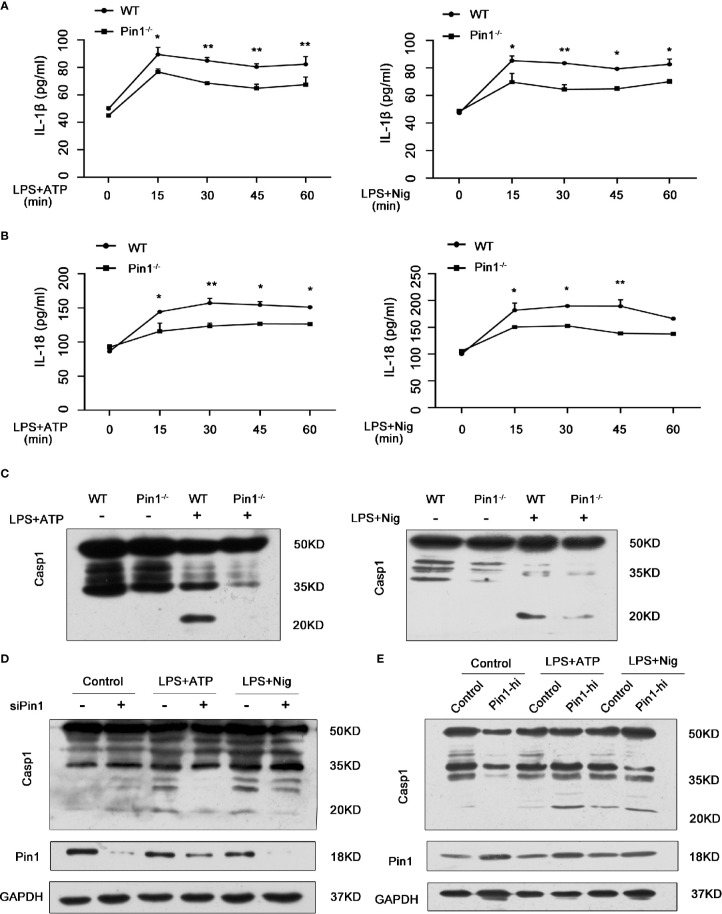
Caspase-1 activation and IL-1β and IL-18 secretion in macrophages were inhibited in Pin1^-/-^ mice. **(A, B)** WT and Pin1^-/-^ bone marrow-derived macrophages (BMDMs) were primed with 500 ng/ml lipopolysaccharide (LPS) for 4 h and stimulated with ATP or nigericin for different lengths of time, supernatants were analyzed for IL-1β and IL-18. **(C)** WT and Pin1^-/-^ BMDMs were primed with 500 ng/ml LPS for 4 h and then stimulated with 5mM ATP or 20 mM nigericin for 30 min. Lysates were immunoblotted for caspase-1. **(D)** RAW 264.7 cells transfected with control siRNA or siRNA for Pin1 were primed with 500 ng/ml LPS for 4 h and then stimulated with 5 mM ATP or 20 mM nigericin for 30 min. Lysates were immunoblotted for caspase-1. **(E)** RAW 264.7 cells with high Pin1 expression (Pin1-hi) were primed with 500ng/ml LPS for 4h and then stimulated with 5 mM ATP or 20 mM nigericin for 30 min. Lysates were immunoblotted for caspase-1. The data represent the mean ± SD of one among three biological replicates, with three technical replicates each. *P < 0.05; **P < 0.01, two-way ANOVA **(A, B)**.

According to the function classification, caspase-1 plays an inflammatory role in the Caspase family (27). We want to know whether Pin1 has an effect on the activation of Caspase1 and production of IL-1β and IL-18 in macrophages depending on caspase-1 activation. Thus, we treated LPS-primed WT and Pin1^-/-^ bone marrow-derived macrophages (BMDMs) with ATP or Nigericin (abbreviated Nig). We found that IL-18 and IL-1β cytokine secretion induced by LPS were reduced during ATP or Nig treatment but not in a time-dependent manner (**Figures 2A, B** **)**. ATP or Nig stimulation was used for 30 min for the following experiment. BMDMs from Pin1^-/-^ mice showed lower cleavage of caspase-1, it indicated that Pin1 knockout inhibited caspase-1 activation (**Figure 2C**). The same results were also found in RAW 264.7 cells (**Figure 2D**). While Pin1 was overexpressed in RAW 264.7 cells, immunoblotting showed increased cleavage of Caspase1, which suggested that Pin1 may promote the activation of Caspase1 (**Figure 2E**).

### Pin1 Regulates NLRP3 Expression and Transcription in Macrophages

**Figure 3:**
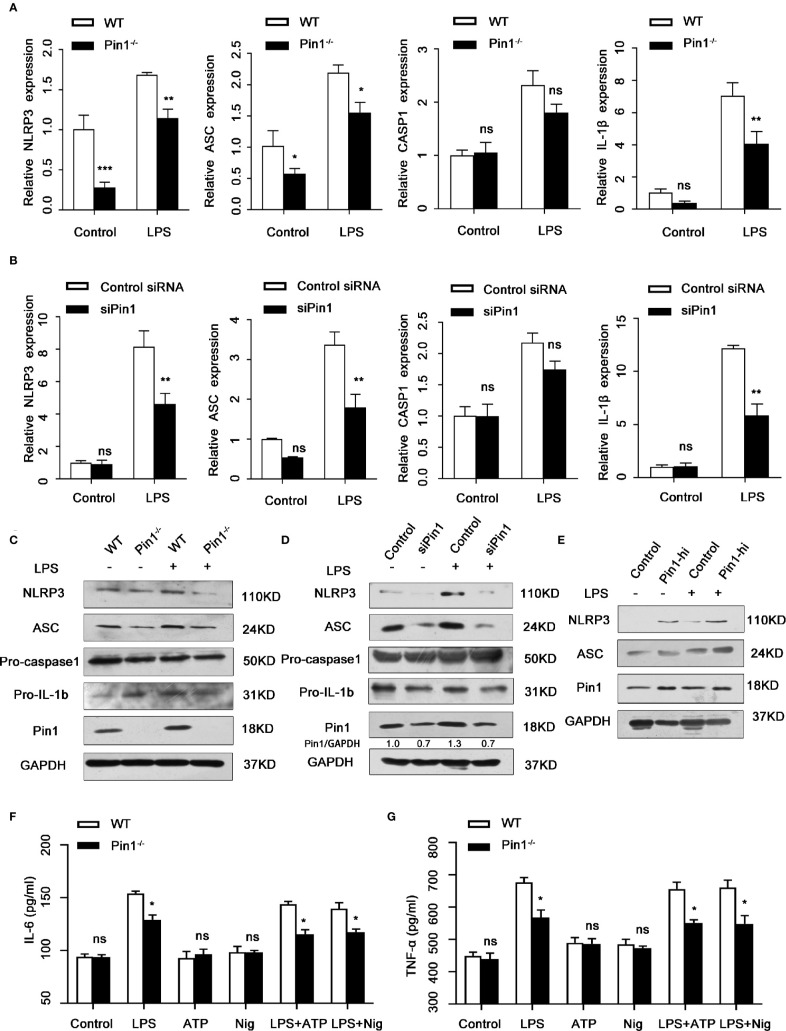
Pin1 regulates NLRP3 expression and transcription in macrophages. **(A)** *Nlrp3*, *Asc*, *Casp1*, and *IL-1β* mRNA levels in LPS-treated WT and Pin1^-/-^ bone marrow-derived macrophages (BMDMs). **(B)** *Nlrp3*, *Asc*, *Casp1*, and *IL-1β* mRNA levels in lipopolysaccharide (LPS)-treated RAW 264.7 cells transfected with control siRNA or siRNA for Pin1. **(C)** IL-1b and NLRP3 inflammasome protein levels in LPS-treated WT and Pin1^-/-^ BMDMs after 4 h. **(D)** IL-1b and NLRP3 inflammasome protein levels in LPS-treated RAW 264.7 cells transfected with control siRNA or siRNA for Pin1 after 4h. **(E)** RAW 264.7 cells with high Pin1 expression (Pin1-hi) were primed with 500 ng/ml LPS for 4h, lysates were immunoblotted for NLRP3 and ASC. **(F, G)** WT and Pin1^-/-^ BMDMs were treated without stimulation (control) or with LPS (500ng/ml), ATP (5mM) or nigericin (20mM) alone, LPS for 4 h and 5 mM ATP or 20 mM nigericin for 30 min. The secretion of IL-6 and TNF-α in culture supernatant were analyzed. The data represent the mean ± SD of one among three biological replicates, with three technical replicates each. *P < 0.05; **P < 0.01; ns, no significance; two-way ANOVA **(A, B, F, G)**.

The activation of Caspase1 is usually regulated by NLRP3 to form the canonical NLRP3 inflammasome complex, we wonder whether Pin1 also affected the expression of NLRP3 besides Caspase1 activation and secretion of the inflammatory factor IL-18 and IL-1β. We first analyzed that the mRNA levels of *Nlrp3*, *Asc*, *Casp1*, *IL-1β* in WT and Pin1^-/-^ bone macrophages in response to LPS. The expression of these genes was significantly decreased in the Pin1^-/-^ group (**Figure 3A**). The protein expression of NLRP3 and ASC was also significantly reduced in Pin1^-/-^ BMDM compared with the control group (**Figure 3C**). Similarly, mRNA levels and protein expression of NLRP3 inflammasome in RAW 264.7 cells transfected with siPin1 were detected and the same results were obtained (**Figures 3B, D** **)**. The expression of NLRP3 and ASC increased in RAW 264.7 cells overexpressing Pin1 (**Figure 3E**). In addition, we also tested the secretion of IL-6 and TNF-α in Macrophage-related cytokines under different factors (**Figures 3F, G** **)**. The results showed that the secretion of IL-6 and TNF- α could not be stimulated when only ATP or Nig was used. Compared with the control group, the Pin1 knockout group had a significant decrease under stimulation. These results suggest that Pin1 is involved in LPS-induced activation of the NLRP3 inflammasome in macrophages.

### Pin1 Deficiency Prevents Caspase-1-Mediated Cleavage of GSDMD

**Figure 4:**
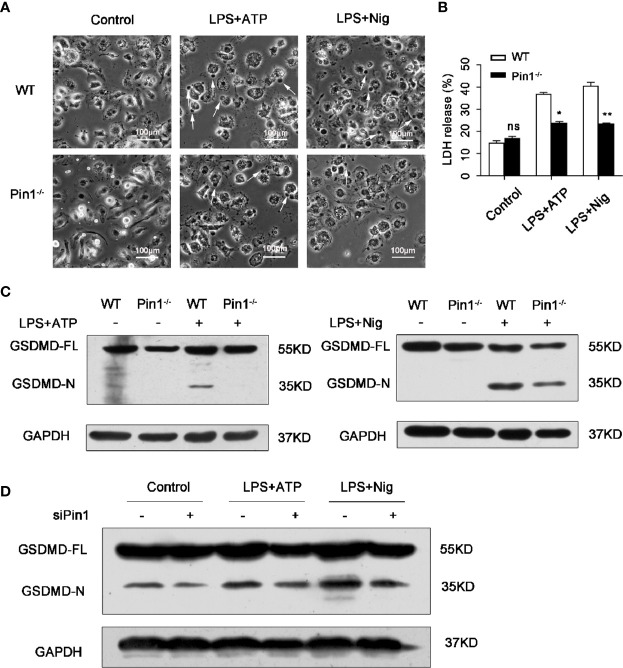
Pin1 deficiency prevents caspase-1-mediated cleavage of gasdermin D (GSDMD). **(A)** Imaging assay of pyroptosis in WT and Pin1^-/-^ bone marrow-derived macrophages (BMDMs) treated with 5 mM ATP or 20 mM nigericin. **(B)** BMDMs were primed with 500 ng/ml LPS for 4 h and stimulated with ATP or nigericin for 30 min and supernatants were analyzed for lactate dehydrogenase (LDH). **(C)** Immunoblotting with mouse antibody for GSDMD in WT and Pin1^-/-^ BMDMs treated with ATP or nigericin. **(D)** Immunoblotting with mouse antibody for GSDMD in RAW 264.7 cells transfected with control siRNA or siRNA for Pin1 treated with 5 mM ATP or 20 mM nigericin for 30 min. All data are representative of three independent experiments. The data represent the mean ± SD of one among three biological replicates, with three technical replicates each. *P < 0.05; **P < 0.01; ***P < 0.001; ns, no significance; two-way ANOVA **(B)**.

To further investigate the role of Pin1 in pyroptosis induced by inflammatory activation. Pyroptosis morphology were observed in WT and Pin1-knockout BMDMs treated with LPS and ATP or Nig. The result showed that cell death reduced in Pin1 KO BMDMs compared with WT BMDMs (**Figure 4A**).

The dying cells show a typical pyroptosis state, and pathological manifestations were cell swelling and membrane rupture (8, 13). We also tested the expression of lactate dehydrogenase (LDH), which is an enzyme released through the broken membrane after cell lysis and death. The results showed that compared with the control group, the release of LDH in the Pin1^-/-^ group was reduced. (**Figure 4B**). In BMDMs treated with LPS and ATP or nigericin, GSDMD was cleaved, which mean that GSDMD was activated and pyroptosis occurred. We found that the cleavage products of GSDMD were significantly reduced in Pin1-deficient cells compared to the control group (**Figure 4C**). Similarly, a reduction in GSDMD cleavage products was also detected in RAW 264.7 cells with Pin1 knockdown (**Figure 4D**). These data indicated that Pin1^-/-^ inhibits GSDMD cleavage-induced pyroptosis.

### Pin1 Regulates NLRP3 Inflammasome Expression by Promoting Phosphorylation of P38 MAPK Pathway

**Figure 5:**
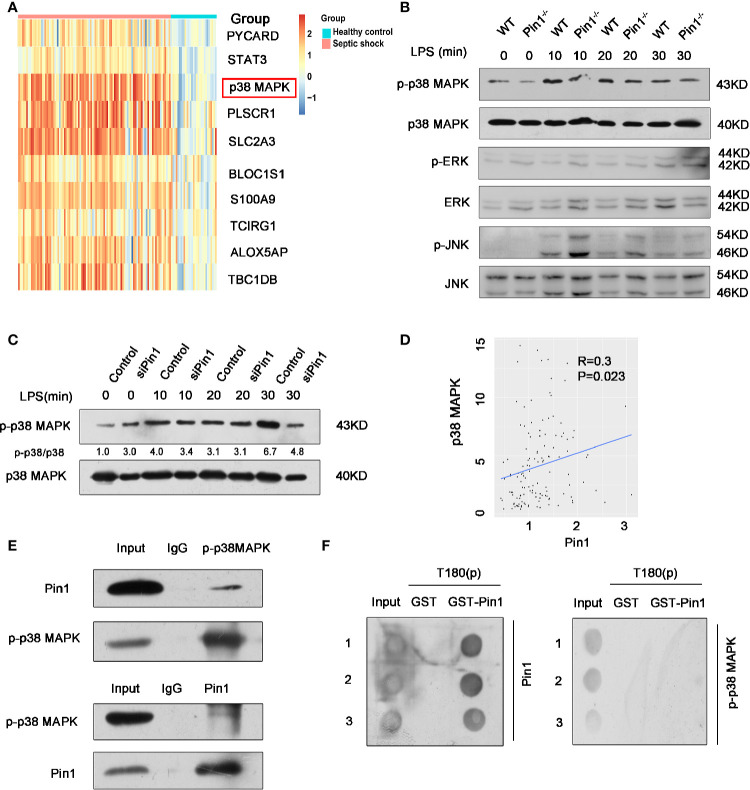
Pin1 regulates NLRP3 inflammasome expression by promoting phosphorylation of p38 MAPK pathway. **(A)** Analysis of one published septic shock clinical data set (GSE26440), revealed that p38 MAPK expression is upregulated in septic shock. **(B)** Immunoblot analysis for activation of p38 MAPK, ERK, and JNK in cell lysates from WT and Pin1^-/-^ bone marrow-derived macrophages (BMDMs) and stimulated with LPS (500 ng/ml) for 0, 10, 20, and 30 min. **(C)** Immunoblot analysis for activation of p38 MAPK in RAW 264.7 cells transfected with control siRNA or siPin1 for 0, 10, 20, and 30 min. **(D)** Correlation analysis between Pin1 and p38 MAPK in GSE26440. **(E)** BMDMs were collected to perform co-immunoprecipitation with p-p38 MAPK or Pin1. **(F)** Dot blot analysis for detecting the interaction between Pin1 and p38 phosphopeptide (T180(P)). The numbers 1,2,3 represent three repetitions.

We have previously demonstrated that Pin1 responds to LPS stimulation through NLRP3 inflammasomes in macrophages. We next investigated the mechanism by which Pin1 regulates the NLRP3 inflammasome. Based on Pin1 specificity in identifying pSer/Thr-Pro motif, we detected the upstream regulatory factors of NLRP3 inflammasome, we found that p38 MAPK was markedly upregulated in the clinical datasets GSE26440 (**Figure 5A**). We then analyzed the effects of Pin1 knockout on the activation of MAPKs, including p38 MAPK, extracellular signal-regulated kinases (ERK) and Jun-amino-terminal kinase (JNK) in macrophages. Western blot showed that phosphorylation levels of p38 MAPK decreased in Pin1^-/-^ macrophages compared with controls, while phosphorylation levels of ERK, JNK showed few significant changes (**Figure 5B**). We also performed immunoblotting in RAW 264.7 cells and found that the expression of p-p38 MAPK in the Pin1 knockdown group was significantly reduced when LPS stimulated for 30 min (**Figure 5C**). We found from a published septic shock clinical data set (GSE26440) that there was a positive correlation between Pin1 and p38 MAPK (**Figure 5D**). This suggests that Pin1 may promote the phosphorylation of the p38 MAPK pathway to regulate NLRP3 inflammasome activation.

To further prove that Pin1 regulates NLRP3 inflammasomes by acting on p38 MAPK signaling pathway, co-immunoprecipitation of p-p38 MAPK and Pin1 was performed. Co-immunoprecipitation analysis showed that Pin1 was bound to p-p-p38 MAPK in BMDMs (**Figure 5E**), which implied that the p-p38 MAPK may be a substrate of Pin1. We want to know whether the interaction between Pin1 and p-p38 MAPK is a direct binding, so we conducted a GST-pulldown experiment, and the results showed that Pin1 cannot directly bind to p-p38 MAPK *in vitro* (**Figure 5F**). This result suggests that Pin1 may affect p-p38 MAPK through kinases or other proteins. Together, these results suggest that Pin1 serves as a crucial effect in NLRP3-mediated inflammation by regulating signaling pathway of p38 MAPK.

### Pin1 Knockdown Down-Regulated NLRP3 Inflammasome in Human Septic Shock

**Figure 6:**
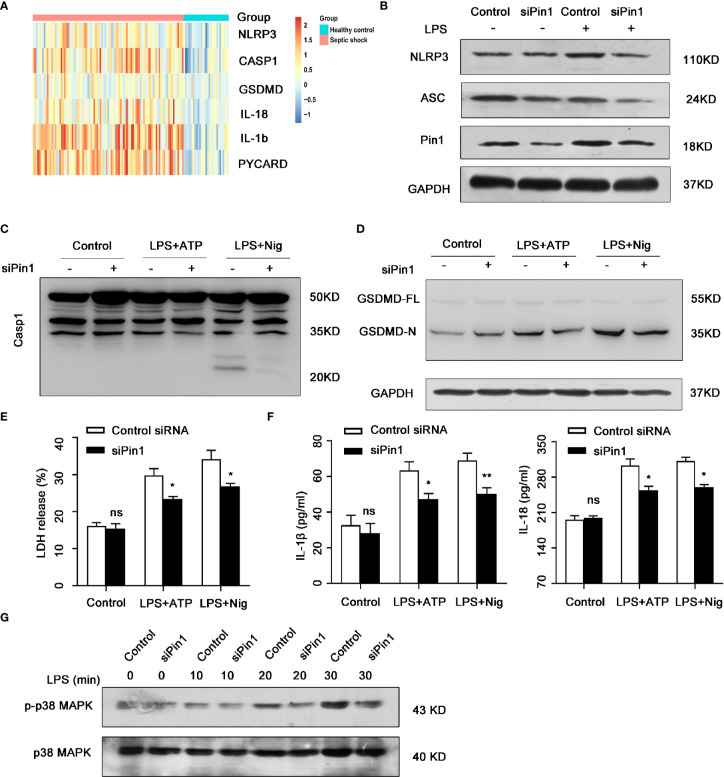
Pin1 knockdown down-regulated NLRP3 inflammasome in human septic shock. **(A)** Analysis of inflammatory genes in published clinical datasets GSE26440. **(B)** PMA-differentiated THP-1 cells were transfected with control siRNA or siPin1 followed by stimulation with 500 ng/ml lipopolysaccharide (LPS) for 4 h. Cell extracts were immunoblotted for NLRP3 inflammasomes. **(C)** PMA-differentiated THP-1 cells were transfected with control siRNA or siPin1 followed by stimulation with 500 ng/ml LPS for 4 h and treatment with 5 mM ATP or 20 mM nigericin for 30 min. Immunoblot was performed for caspase-1 activation. in cell extracts. **(D)** PMA-differentiated THP-1 cells were transfected with control siRNA or siPin1 followed by stimulation with 500 ng/ml LPS for 4 h and treatment with 5 mM ATP or 20 mM nigericin for 30 min. Immunoblot was performed for GSDMD cleavage in cell extracts. **(E, F)** PMA-differentiated THP-1 cells were transfected with control siRNA or siPin1 followed by stimulation with 500 ng/ml LPS for 4 h and treatment with 5 mM ATP or 20 mM nigericin for 30 min. Supernatants were analyzed for LDH, IL-1β, and IL-18. **(G)** Immunoblot analysis for activation of p38 MAPK in PMA-differentiated THP-1 cells transfected with control siRNA or siPin1 and stimulated with LPS (500 ng/ml) for 0, 10, 20, and 30 min. The data represent the mean ± SD of one among three biological replicates, with three technical replicates each. *P < 0.05; **P < 0.01; ns, no significance; two-way ANOVA **(E, F)**.

We further analyzed the expression of inflammation-associated protein in human septic shock (**Figure 6A**). Next, we use LPS-treated PMA-differentiated THP-1 cells that transfected siPin1 and siRNA control, the result showed that NLRP3 and ASC protein levels were downregulated in Pin1 knockdown group compared with control group (**Figure 6B**). We also treated LPS-primed PMA-differentiated THP-1 cells with ATP or Nig and found that the Pin1 knockdown reduced caspase-1 activation and cleavage of GSDMD (**Figures 6C, D** **)**. In addition, the expression of IL-18,IL-1β and LDH also decreased in the Pin1 knockdown group (**Figures 6E, F** **)**. In THP-1 cells, Pin1 knockdown also reduced the expression of p-p38 MAPK (**Figure 6G**). These data also show that Pin1 plays an important inflammatory effect in human septic shock.

**Figure 7:**
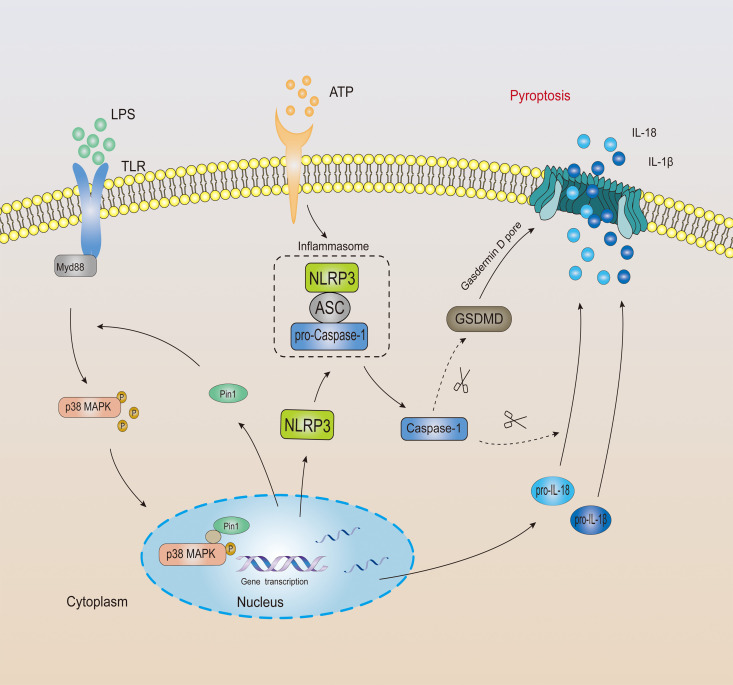
The mechanistic model of Pin1 mediated inflammasome activation and pyroptosis.

In general, Pin1 plays an important role in NLRP3-mediated caspase-1 activation and cleavage of GSDMD in mouse and human (**Figure 7**).

## Discussion

In this article, we revealed the role of Pin1 in LPS-induced septic shock. Pin1 is the only small molecule enzyme in the body that can recognize threonine/serine-proline motifs. The isomerization of a large number of phosphorylated proteins can regulate many cell activities, leading to the occurrence of diseases such as cancer, Alzheimer’s disease, and autoimmune diseases (22, 26). However, the role of Pin1 in inflammatory diseases such as septic shock remains undefined. Here, we reported that Pin1 deficiency relieves LPS-induced septic shock and organ damage. Our results showed that Pin1 is critical for NLRP3-mediated caspase-1 activation and promotes the cleavage of GSDMD as well as IL-1β and IL-18 secretion *in vivo* or *in vitro*. Furthermore, Pin1 mediates IL-6 or TNF-α production and NLRP3, ASC, caspase-1 and IL-1β expression by binding to p38 MAPK protein.

NLRP3 inflammasome mediates the occurrence of many inflammatory diseases, including septic shock (12, 15). In this process, NLRP3 mediates the activation of Caspase1, the activated caspase1 can activate the cleavage of downstream target GSDMD and Pro-IL-1β and Pro-IL-18 secreted by macrophages (13, 28). We found that knock out Pin1 can decrease the mortality of mice infected with LPS and alleviate the damage to the internal organs induced by LPS. Next, we further studied whether the occurrence of this phenomenon is carried out by affecting the expression of NLRP3 inflammasome. We performed the same treatment on BMDMs and RAW 264.7 cells, as we expected, it was that knockout/knockdown Pin1 can reduce NLRP3-mediated caspase-1 activation in response to LPS and ATP or Nigericin. Moreover, other components of the inflammasome such as NLRP3 and ASC also declined significantly. Besides, we analyzed from the published clinical septic shock dataset that the expression of NLRP3 inflammasome and related gene are lower in the healthy, we also found consistent results in PMA-induced THP1 cells.

Caspase-1 and caspases11 (caspase-4 and caspase-5 in humans) can specifically recognize and then cleave gasdermin D (GSDMD), which is identified as the key event in pyroptosis (8, 13). The N-terminal domain of GSDMD can specifically bind to membrane lipids, and after binding to membrane lipids, the N-terminal domains of GSDMD- oligomerize to form pores of about 12–14 nm in inner diameter (13, 28–31). By directly observing the cell morphology of pyroptosis and detecting the related proteins, we found that the degree of pyroptosis was significantly reduced after the absence of Pin1.

Excessive inflammation response is the cause of many human diseases (13). Many protein kinases include JNKs, p38 MAPKs, and ERKs that are activated in response to various stresses (22, 32). Among them, the p38 MAPK pathway, is usually involved in the stress response and inflammation in the body (29, 33). It has been reported that the p38 MAPK pathway is closely related to LPS-induced septic shock (1, 8, 34, 35). We found from the Gene Expression Omnibus database that the expression of p38 MAPK increased in patients with septic shock. At the same time, we also found a certain positive correlation between Pin1 and p38 MAPK in the database, which suggests that Pin1 may regulate the expression of NLRP3 inflammasomes through the p38 MAPK pathway. To prove this hypothesis, we first performed co-immunoprecipitation analysis of Pin1 and p-p38 MAPK. We found that Pin1 and p-p38 MAPK bind in BMDMs. Then, we further analyzed this interaction through the GST-pulldown experiment, and the results showed that Pin1 cannot directly bind to p-p38 MAPK (T180 site). Pin1 is likely to affect the phosphorylation of p38 MAPK through kinases or other proteins. Recent studies have found that Pin1 regulates the phosphorylated conformation of proteins between two different structures depending on specific targets and local structural constraints. This processes are controlled by pin1-catalyzed phosphorylation changes rather than the initial phosphorylation itself (22). It is likely that Pin1 binds to p-p38 MAPK and forms a complex. When encountering an external stimulus, Pin1 recognizes and accelerates the phosphorylation process. (**Figure 7**). Together, these results suggest that Pin1 affects the expression of inflammatory proteins through the p38 MAPK signaling pathway.

However, it is interesting that Akiyama et al. believe that Pin1 has a protective effect on LPS-induced shock (36). Pin1^-/-^ mice were originally created by Uchida’s laboratory in 1999 (37). Recently, it has been reported that there is premature aging phenomenon in Pin1^-/-^ mice (38), the same phenomenon has been observed in our laboratory. The difference in mouse age and the dosage of LPS, which may be the reason for our different results. In fact, except for some cancers, Pin1 also plays an important role in asthma development and in the response to microbial infection besides rheumatoid arthritis (RA), Systemic Lupus Erythematosus (SLE) and nonalcoholic steatohepatitis (NASH) (39–44), suggesting that Pin1 is closely related to certain inflammatory reactions. Our study offers clues for understanding septic shock pathogenesis and might impact on the future diagnosis and treatment of patients with inflammatory disease. However, further clinical research remains to be investigated.

## Data Availability Statement

The datasets presented in this study can be found in online repositories. The names of the repository/repositories and accession number(s) can be found below: https://www.ncbi.nlm.nih.gov/geo/, GSE26440.

## Ethics Statement

The animal study was reviewed and approved by Tianjin Medical University.

## Author Contributions

RD and YD designed the experiments and wrote the manuscript. RD, ZX, GF, NZ, CW, and GL performed the experiments and data analysis. ZX and YD revised the manuscript. All authors contributed to the article and approved the submitted version.

## Funding

This study was supported by the Science &Technology Development Fund of Tianjin Education Commission for Higher education, key projects through grant nos 2018ZD05, the Natural Science Foundation of Tianjin through grant nos 16JCYBJC24600, and the National Natural Science Foundation of China through grant nos. 81541032.

## Conflict of Interest

The authors declare that the research was conducted in the absence of any commercial or financial relationships that could be construed as a potential conflict of interest.
